# A Nonlinear Fitting Method Provides Strong Support for Geometric Series of Stomatal Area in 12 Magnoliaceae Species

**DOI:** 10.3390/plants14060893

**Published:** 2025-03-12

**Authors:** Chunxiu Yan, Peijian Shi, Weihao Yao, Kexin Yu, Ülo Niinemets

**Affiliations:** 1 National Key Laboratory of Smart Farm Technologies and Systems, College of Plant Protection, Northeast Agricultural University, Harbin 150030, China; yanchunxiu@neau.edu.cn; 2 Southern Modern Forestry Collaborative Innovation Center, College of Ecology and Environment, Nanjing Forestry University, #159 Longpan Road, Nanjing 210037, China; whyao@njfu.edu.cn (W.Y.); kxyu@njfu.edu.cn (K.Y.); 3 Institute of Agricultural and Environmental Sciences, Estonian University of Life Sciences, Kreutzwaldi 1, 51006 Tartu, Estonia

**Keywords:** common ratio, first term, Magnoliaceae, nonlinear regression, stomatal size

## Abstract

Stomatal pore area and density determine the capacity for gas exchange between the leaf interior and the atmosphere. Stomatal area is given by the profile formed by two guard cells, and the cumulative stomatal area characterizes the area of leaf surface occupied by stomata. The areas of all stomata captured in a micrograph are sorted in ascending order to form a sequence, which is referred to as a sequence of stomatal area here. In total, 360 leaves of 12 Magnoliaceae species with 30 leaves for each species were sampled. For each leaf, two 662 μm × 444 μm fields of view (micrographs) of stomata were captured on the right leaf width axis. In each micrograph, the length and width of each stoma were measured, and the area of the stoma was determined using the product of stomatal length and width multiplied by a proportionality coefficient. Stomatal area sequences of Magnoliaceae in the constant field of view were found to follow a geometric series (GS). Prior studies estimated the common ratio of the GS as the mean of the quotients of any two adjacent terms, and estimated the first term as the mean of the first terms (i.e., the smallest stomatal area) represented by the quotient of each term and the estimated common ratio to a power of the order of the term minus 1, which is referred to as Method-1. However, it produced large prediction errors for some stomatal area sequences. In the present study, the nonlinear regression was used to fit the stomatal area sequences using the common ratio and the first term as two model parameters (Method-2). We compared the two methods using the mean absolute percent error (MAPE, ≤5% considered as a good fit) values of the 720 stomatal micrographs from the 12 Magnoliaceae species. The goodness of fit of Method-2 was better than that of Method-1 (52.4% MAPE values were ≤5% for Method-1 and 99.6% for Method-2). There were significant variations in the estimated common ratios, as well as the estimated first terms and the MAPE values across the 12 Magnoliaceae species, but overall, the interspecific differences in the MAPE values were small. We conclude that the GS hypothesis for the stomatal area sequences of the 12 Magnoliaceae species was further strengthened by the new method. This method further provides a valuable approach for the calculation of total stomatal area per unit leaf area.

## 1. Introduction

Stomata, microscopic pores predominantly located on the epidermal surfaces of leaves, serve as critical gateways for gas exchange in terrestrial plants [1]. Each stoma consists of a pair of specialized guard cells that dynamically regulate pore aperture in response to environmental cues such as light, humidity, and CO_2_ concentration, thereby balancing CO_2_ uptake for photosynthesis with water loss via transpiration [2,3]. This dual role positions stomata at the nexus of plant productivity and hydraulic efficiency, making their structural and functional traits central to understanding plant adaptation to environmental stressors [4]. Stomatal size, density, and responsiveness to environmental drivers directly influence the efficiency of gas exchange, with variations in these traits reflecting evolutionary compromises between carbon assimilation and water conservation [2,5,6]. In addition, two factors also play distinct roles in influencing the efficiency of gas exchange: (i) stomatal spacing distance (where wider spacing enhances gas exchange) [7], and (ii) stomatal distribution between the two leaf surfaces (where a more balanced arrangement promotes gas exchange) [8].

Stomatal conductance correlates with photosynthetic capacity [9,10]. Maximum stomatal conductance (*g*_smax_) represents the theoretical upper limit of gas diffusion through fully open stomata under optimal environmental conditions, such as saturating light, high humidity, and adequate water supply. Physiologically, *g*_smax_ is determined by anatomical features, including stomatal size (length and width of the pore and guard cells), stomatal density (i.e., the number of stomata per unit leaf area), the pore area per stomatal area, and the internal architecture of the stomatal complex (e.g., substomatal cavity depth) [11,12,13]. Larger stomata generally exhibit higher *g*_smax_ due to a greater pore area, but their slower response kinetics to environmental fluctuations can reduce overall water use efficiency under variable conditions. Conversely, smaller stomata enable faster aperture adjustments, minimizing transient water loss during drought, albeit at the cost of reduced peak CO_2_ uptake [2,6]. Stomatal density amplifies these effects: high-density configurations compensate for small individual pore sizes by increasing the total pore area, whereas lower stomatal densities prioritize hydraulic safety over carbon gain [11]. The relationship between stomatal size and density is frequently characterized by a negative correlation across plant taxa, a phenomenon attributed to developmental constraints on epidermal patterning [11]. The “one-cell-spacing rule” posits that stomatal precursor cells inhibit neighboring cells from differentiating into stomata, creating a spatial trade-off where larger stomata occupy more epidermal space, thereby limiting density [13,14]. Although stomatal size and density play an important role in constraining *g*_smax_, the spatial and size distribution features, including dispersion of stomata and size variation, have been largely neglected in prior studies.

The Magnoliaceae family is an ancient lineage of flowering plants and constitutes an interesting model system characterized by early-evolved paracytic stomata [15]. The Magnoliaceae genera, e.g., *Liriodendron*, *Magnolia*, and *Michelia*, exhibit similar stomatal morphology formed by two kidney-shaped guard cells. The outer profiles of the stomata were found to be hyper-ellipses (a group of smooth geometries between ellipses and rectangles) [16]. The stomatal area (*A*), defined as the area of the outer profile by two guard cells, is proportional to the product of stomatal length (*L*) and width (*W*), i.e., the length and width of the outer profile formed by two guard cells, within and across species [17]:

(1) A=kLW,

where *k* is the proportionality coefficient, estimated to be 0.8071 at the Magnoliaceae family level. Equation (1) was first proposed to describe the proportional relationship between leaf area and the product of leaf length and width [18,19,20,21,22].

Recently, Yan et al. [23] hypothesized that the stomatal area sequence in a micrograph follows a geometric series (denoted as GS, i.e., a series in which each term is equal to the former term multiplied by a constant named the common ratio). To test this hypothesis, based on each of 720 stomatal micrographs (size of the field of view: 662 μm × 444 μm) of 12 Magnoliaceae species with 60 micrographs for each species, they used the mean of the quotients of the adjacent stomatal areas as the estimate of the common ratio of GS, and used the mean of the quotients of each term of GS and the estimated common ratio to a power of *i* − 1 as the estimate of the first term of GS, where *i* represents the order of a term in the GS in ascending order (*i* ≤ *n*, where *n* represents the total number of stomata in a micrograph). Because the sum of the GS is more important in determining stomatal area per unit leaf area [23], the formula of the sum of the first *j* terms (*j* ≤ *n*) was used to fit the observed total area of the *j* stomata in a micrograph. However, in assessing the goodness of fit of the method, there are two problems in [23]: (i) the estimate of the common ratio and that of the first term of GS likely did not result in the global optimization by minimizing the residuals between the observed and predicted values, and (ii) the coefficient of determination (*r*^2^) was used to reflect the goodness of fit; this is not necessarily suitable for the nonlinear regression [24]. For a stomatal area sequence in ascending order, represented by {*A_i_*} (*i* = 1, 2, 3, …, *n*, where *n* represents the total number of stomata in a micrograph), we can consider the order of each term of a GS (i.e., i) as the predicator, the area of each stoma (i.e., *A_i_*) as the response variable, and the common ratio (*q*) and the first term (*A*_1_) as two parameters to be estimated, given that $A_{i}=A_{1}q^{i-1}$ (i.e., the formula of the general term of a GS). In this case, we can estimate the common ratio and the first term using the nonlinear regression. In the present study, we tested the validity of the nonlinear regression compared by the prior method in [23] using the stomatal area sequences of 720 micrographs.

## 2. Materials and Methods

### 2.1. Plant Sampling and Data Acquisition of Stomatal Area

Six *Magnolia* species (*Magnolia biondii* Pamp., *Magnolia denudata* Desr., *Magnolia grandiflora* L., *Magnolia × soulangeana* Soul.-Bod., *Magnolia stellata* (Sieb. & Zucc.) Maxim., and *Magnolia zenii* W.C. Cheng) and six *Michelia* species (*Michelia cavaleriei* var. *platypetala* (Hand.-Mazz.) N.H. Xia, *Michelia chapensis* Dandy, *Michelia figo* (Lour.) Spreng., *Michelia foveolata* Merr. ex Dandy, *Michelia martini* (H. Lév.) Finet & Gagnep. ex H. Lév., and *Michelia maudiae* Dunn.), grown in Nanjing, Jiangsu Province, China, were sequentially coded as species from 1 to 12, respectively, to conveniently compare the variation in the distribution features of stomatal area across these species. The plants were grown at two adjacent sites (2.6 km apart), the Nanjing Forestry University Xinzhuang Campus and the Nanjing Botanical Garden, Chinese Academy of Sciences. Both sites had nearly identical climates and garden management regimes, reducing the influence of environmental conditions on stomatal morphology. More than 40 healthy, mature, and intact leaves were sampled from the lower canopy of each tree (i.e., the bottom third of each tree crown) between late June and mid-August 2022. Stomatal features (*L* and *W*) were ultimately measured in 30 leaves in each species (Table 1 in [23] for details of leaf sampling).

**Figure 1:**
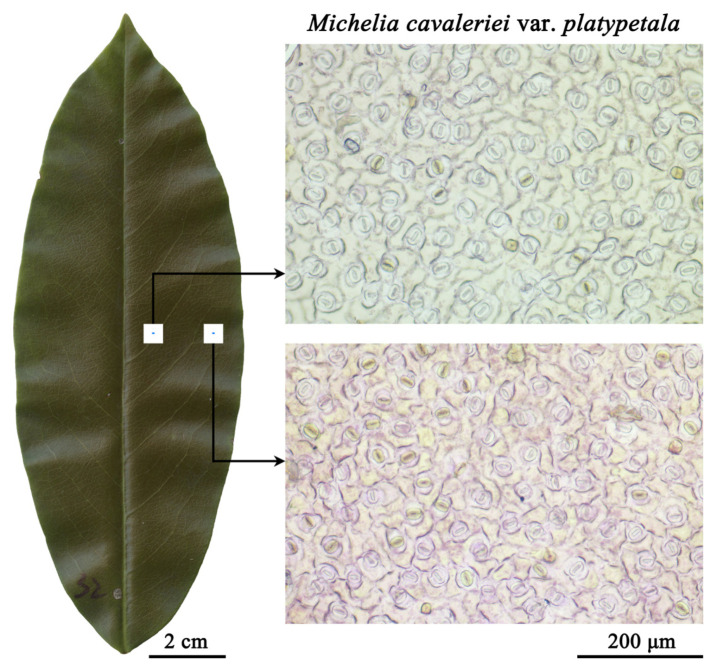
Sampling positions of the two quadrats for a leaf example. One 0.5 cm × 0.5 cm quadrat is near the midrib, and another quadrat is near the right leaf margin. For each quadrat, a 662 μm × 444 μm field of view was photographed. For each of the remaining 359 leaves from the 12 Magnoliaceae species, the same sampling method was adopted.

On the right side of each leaf lower surface (i.e., the abaxial side of each leaf), two 0.5 cm × 0.5 cm quadrats were sampled along the leaf width axis (i.e., the straight line perpendicular to the leaf length axis connecting leaf apex and leaf base). The centers of the two quadrats and their midpoint approximately separate the right side on half leaf width into four equidistant segments. One quadrat was near the midrib and the other quadrat near the lamina right margin (Figure 1). We used the segregation method to acquire the stomatal micrographs; detailed steps of the segregation method were listed in [17,25]. A 662 μm × 444 μm field of view from the center of each lamina quadrat was photographed using an optical microscope (NE-620; Nanjing Yongxin Optics Co., Ltd., Nanjing, China) and ImageView software (version 4.11.17864; Shanghai Pooher Electro-Optical Co., Ltd., Shanghai, China that was installed on a desktop Lenovo XiaoXinAir 15ITL 2021 computer, Lenovo Group Limited, Hong Kong, China). Then, the micrographs were exported and saved as TIF files at 72 ppi (pixels per inch) resolution with 5440 × 3648 pixels. The ImageJ software (version 1.53e, https://imagej.net/ij/ [accessed on 23 January 2022]) was used to measure stomatal length (*L*, representing the maximum distance on the outer profile formed by two guard cells) and stomatal width (*W*, representing the maximum width of the outer profile formed two guard cells). The raw data of *L* and *W* of each stoma are provided in Supplementary Data Table S1 in [23]. Using Equation (1), *A* of each stoma was determined.

### 2.2. Methods

To test the validity of the geometric series (GS) hypothesis for the stomatal area distribution per micrograph, we sorted the stomatal areas in ascending order for each micrograph and used the formula for the sum of the first *j* terms (1 ≤ *j* ≤ *n*, where *n* represents the total number of stomata in a micrograph) of a GS (denoted as *S_j_*) to compare the observed cumulative stomatal area from the smallest stoma to the *j*th stoma in the sorted stomatal area sequence for each micrograph.

(2) $$S_{j}=\sum_{u=1}^{j}A_{u}=A_{1}\frac{{\left(1-q\right)}^{j}}{1-q},$$

where *q* represents the common ratio of the GS, and *A*_1_ represents the first term of the GS, i.e., the area of the smallest stoma. In [23,26], *q* was estimated using the mean of the quotients of any two adjacent stomatal areas in a micrograph (denoted as ${\hat{q}}_{1}$), i.e.,

(3) $${\hat{q}}_{1}=\frac{\sum_{i=2}^{n}\left(A_{i}/A_{i-1}\right)}{n-1}.$$

After substituting Equation (3) to Equation (2), the sum of the first *j* terms of the GS (i.e., the cumulative stomatal area from the smallest stoma to the *j*th stoma) can be calculated. Nevertheless, a non-representative abnormal value (too small or too large) of the area of the smallest stoma can cause a large prediction error of Equation (2). To solve this problem, *A*_1_ was estimated as the mean of the predicted values of the first term based on all terms of the GS, using the general term formula of the GS, $A_{i}=A_{1}q^{i-1}$ [23,26]:

(4) $${\hat{A}}_{11}=\frac{1}{n}\sum_{i=1}^{n}\left[A_{i}/{\left({\hat{q}}_{1}\right)}^{i-1}\right],$$

where ${\hat{A}}_{11}$ represents the estimate of the first term of the GS. Since the estimates of the common ratio and the first term of the GS are known, the cumulative leaf area for the first *j* stomata of the sorted stomatal area sequence can be estimated using Equation (2). We referred to this method as **Method-1**.

For a sorted stomatal area sequence in ascending order, each term can be expressed as $A_{i}=A_{1}q^{i-1}$ (*i* = 1, 2, 3, …, *n*) as mentioned above, where *A_i_* and *i* can be regarded as the dependent and independent variables, respectively, and *q* and *A*_1_ can be regarded as the model parameters. In this case, for the *n* pairs of observations of $\left\{i,A_{i}\right\}$ (*i* = 1, 2, 3, …, *n*), the nonlinear least-squares method can be used to fit the observations by minimizing the residual sum of squares using the Nelder-Mead optimization algorithm [27]. The estimates of the common ratio and the first term of the hypothesized GS were represented by ${\hat{q}}_{2}$ and ${\hat{A}}_{21}$. Substituting the estimates of the two parameters to Equation (2), we can calculate the cumulative stomatal area for the first *j* stomata of the sorted stomatal area sequence. We called this method based on the nonlinear regression as **Method-2**.

To assess the goodness of fit of each of the two methods, we calculated the mean absolute percentage error (MAPE) between the observed and predicted cumulative stomatal areas for each micrograph:

(5) $$\mathrm{MAPE}=\frac{1}{n}\sum_{j=1}^{n}\left\{\left|{\hat{S}}_{j}-\sum_{u=1}^{j}A_{u}\right|/\sum_{u=1}^{j}A_{u}\right\}\times 100\%,$$

where ${\hat{S}}_{j}$ represents the predicted cumulative stomatal area for the first *j* stomata (i.e., the sum of stomatal areas from the smallest stoma to the *j*th stoma) in a micrograph using Method-1 or Method-2 based on Equation (2). As a rule of thumb, a ≤ 5% MAPE indicates a good fit [20,22].

Paired *t*-test was used to test whether there was a significant difference in the mean MAPE values between Method-1 and Method-2. Tukey’s honestly significant difference (HSD) test with a 0.05 significance level [28] was used to test the significance of differences in the estimates of the common ratios, the first terms and the MAPE values between any two of the 12 Magnoliaceae species.

All calculations were performed and the figures were constructed using R version 4.2.0 [29], and the nonlinear regression was carried out using the “fitIPEC” function in an R package “IPEC” (version 1.1.0) [30]. The “IPEC” package is used to perform the nonlinear least-square regression based on the Nelder-Mead optimization method [27], and to assess nonlinear models based on some measures of nonlinearity. These measures indicate the adequacy of a local linear approximation of a nonlinear model and its effects on inferences, including calculations of the intrinsic curvature, parameter–effects curvature, Box’s bias and Hougaard’s skewness [31,32,33,34].

## 3. Results

**Figure 2:**
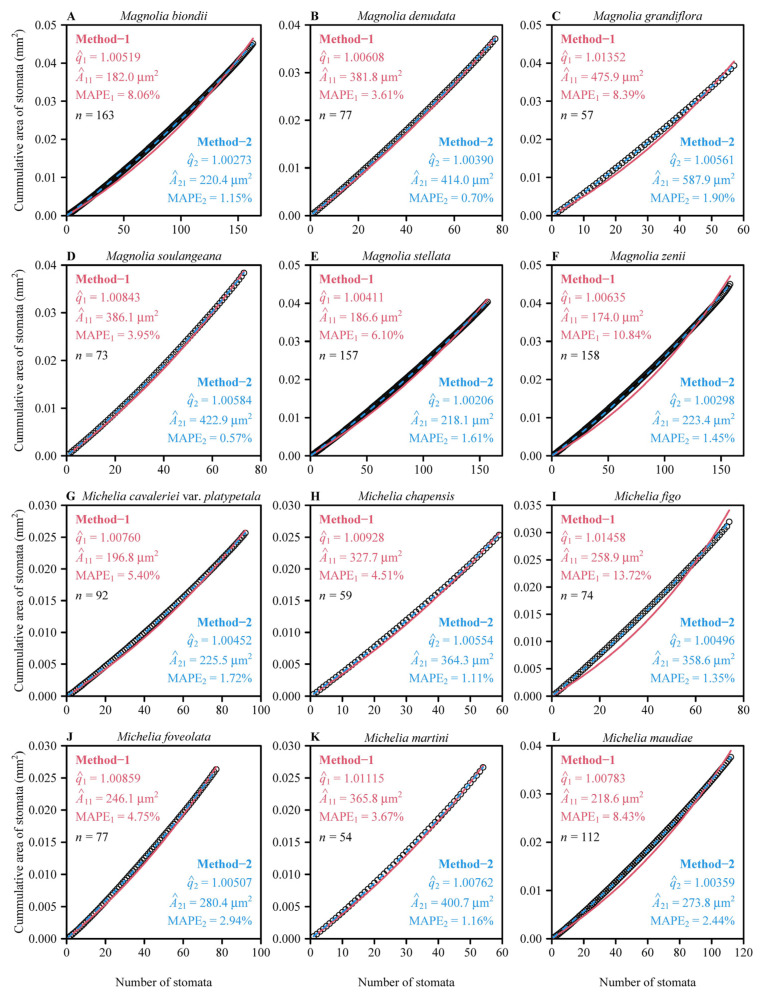
Fitted results of the geometric series to the observed cumulative stomatal area sequences of 12 micrograph examples for individual Magnoliaceae species. Here ${\hat{q}}_{1}$ and ${\hat{A}}_{11}$ represent the estimates of the common ratio and the first term of the stomatal area geometric series for each micrograph using Method-1; ${\hat{q}}_{2}$ and ${\hat{A}}_{21}$ represent the estimates of the common ratio and the first term of the stomatal area geometric series for each micrograph using Method-2; *n* is the total number of stomata for each micrograph; MAPE_1_ and MAPE_2_ are the mean absolute percentage error (MAPE) values between the observed and predicted cumulative leaf stomatal areas for each micrograph from one stoma to the *n* stomata (i.e., between the observed and predicted *S*_1_, *S*_2_, *S*_3_, …, and *S_n_*; Equation (5)) using Method-1 and Method-2. The open circles represent the observations, the red solid line represents the predicted values using Method-1, and the blue dashed line represents the predicted values using Method-2. Panels (**A**–**L**) represent the 12 Magnoliaceae species (see Section 2.1 for details).

**Figure 3:**
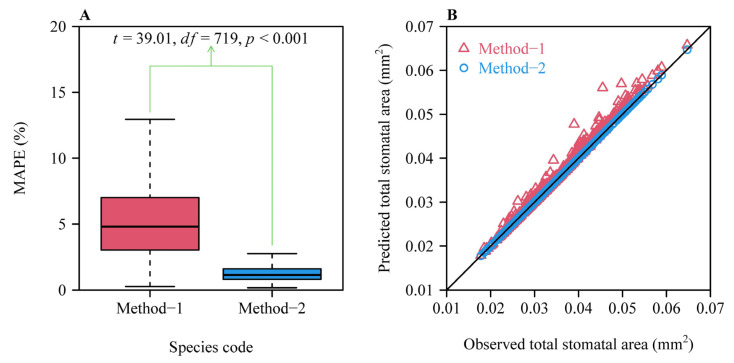
Comparisons of the mean absolute percent error (MAPE) values (**A**), and the observed and predicted total stomatal areas for each of 720 micrographs (**B**) between the two methods. In panel (**A**), each MAPE value quantifies the goodness of fit between the observed and predicted cumulative stomatal areas from one stoma to the *n* stomata in a micrograph (i.e., between the observed and predicted *S*_1_, *S*_2_, *S*_3_, …, and *S_n_*; Equation (5)). In panel (**B**), each symbol represents the observed and predicted total stomatal area for the *n* stomata in a micrograph (i.e., the observed and predicted *S_n_*; Equation (2)). The open triangles (Method-1) apparently deviated from the *y = x* line, differently from the open circles (Method-2).

**Figure 4:**
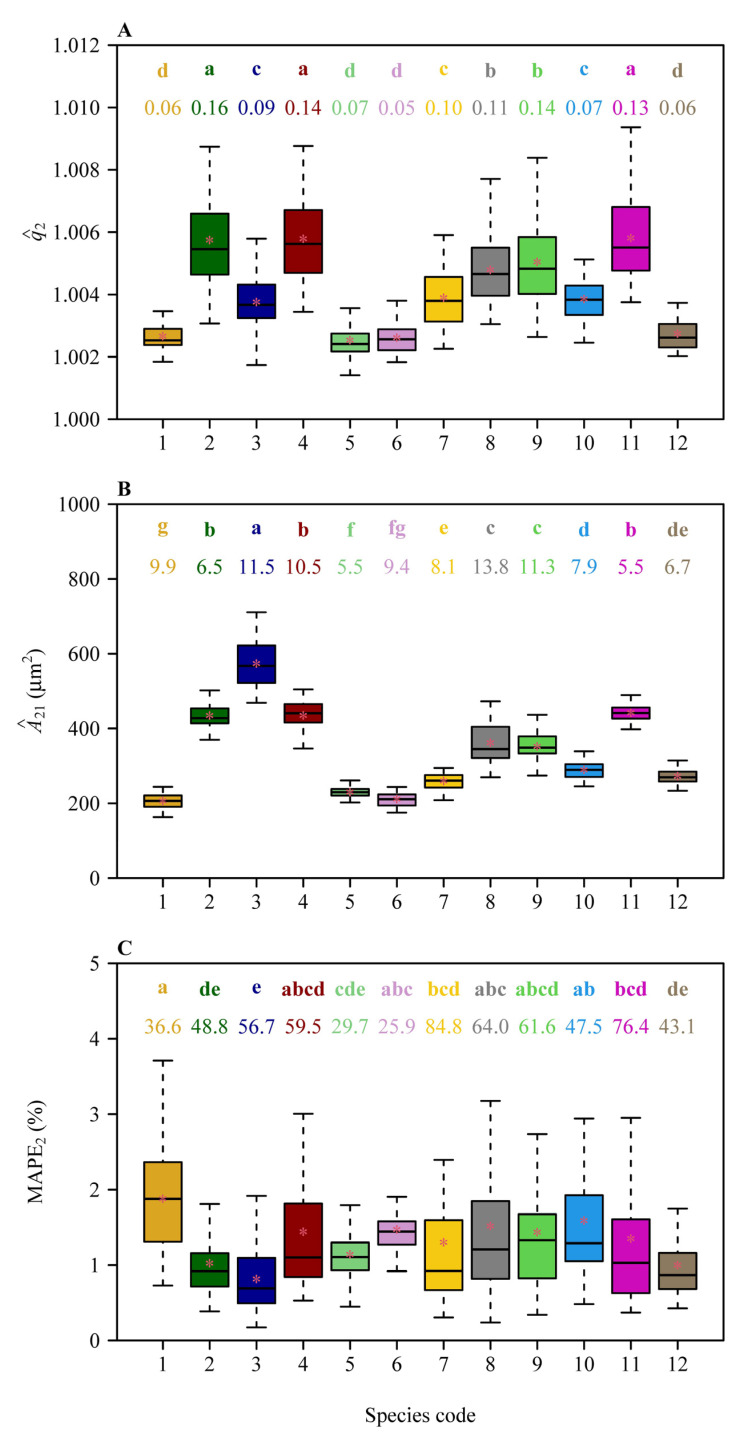
Boxplots of the estimates of the common ratios (**A**), the estimates of the first terms (**B**), and the mean absolute percent errors (**C**) for the 12 Magnoliaceae species with 60 micrographs for each species using Method-2. The upper and low ends of each box represent the 75% and 25% quantiles, respectively. Significant differences among the 12 species are indicated by the lowercase letters in each panel, based on the HSD test (α = 0.05). The numbers below the lowercase letters are the coefficients of variation (%). The horizontal bold lines in the boxes represent the medians, and the asterisks represent the means. Species codes 1−12 represent *Magnolia biondii*, *M. denudata*, *M. grandiflora*, *M. soulangeana*, *M. stellata*, *M. zenii*, *Michelia cavaleriei* var. *platypetala*, *M. chapensis*, *M. figo*, *M. foveolata*, *M. martini*, and *M. maudiae*.

Overall, both methods provided good fits to individual micrograph data in all species, albeit MAPE value was generally greater for Method-1 (Figure 2). There were 377 micrographs (approximately 52.4% of the 720 micrographs) with the MAPE values ≤ 5% for Method-1, whereas there were 717 micrographs (approximately 99.6% of the 720 micrographs) with the MAPE values ≤ 5% for Method-2. There were 646 micrographs (approximately 89.7% of the 720 micrographs) with the MAPE values ≤ 10% for Method-1, whereas there were 100% of the 720 micrographs with the MAPE values ≤ 10% for Method-2. The mean ± standard error of the MAPE values for Method-1 (5.49% ± 3.33%) was significantly greater than that for Method-2 (1.33% ± 0.79%; *t* = 39.01, *df* = 719, *p* < 0.001; Figure 3A). The data indicate that Method-2 provides stronger evidence that the stomatal area sequences of the investigated 720 micrographs from 12 Magnoliaceae species follow a geometric series. The prediction error of the total stomatal area per micrograph, based on Method-2 (with *r*^2^ approximately equaling unity), was smaller than the prediction error based on Method-1 (with *r*^2^ = 0.9892) (Figure 3B). There were significant differences in the estimates of the common ratios, the first terms, and the MAPE values across the 12 Magnoliaceae species (Figure 4).

## 4. Discussion

### 4.1. The Superiority and Limitation of Method-2

The nonlinear regression method (Method-2) directly estimates the common ratio and the first term of the geometric sequence by globally optimizing the sum of squared residuals, avoiding the potential accumulation of errors in the stepwise estimation of Method-1 [32,33]. Method-1 is sensitive to outliers (such as anomalously small stomatal areas), while Method-2, by minimizing the sum of squared residuals, can more robustly adapt to data fluctuations. This improvement indicates that in the fitting of complex biological data, joint parameter optimization is more advantageous than stepwise estimation [27]. Additionally, the high accuracy of Method-2 (99.6% of the mean absolute percentage error (MAPE) values ≤ 5%) supports the hypothesis that the stomatal area sequence strictly follows a geometric series (GS), providing a basis for subsequent theoretical plant physiological models (such as the mathematical laws of stomatal development) and scaling theory of stomatal size [11,13].

Although Method-2 performs well, its applicability still needs further validation. For example, this study is only focused on Magnoliaceae species which have approximately oblong stomata following the hyperellipse equation [16,35], and the stomatal morphology of other families and genera may exhibit different geometries [15]. Additionally, the resolution of the microscopic images (72 ppi) may decrease accuracy of small stomatal areas. In the future, higher-resolution imaging techniques can be used to further reduce systematic errors. The next step could involve combining the molecular mechanisms of stomatal development (such as the cytokinin signaling pathway) to explore the biological causes of the geometric sequence and to explore the application potential of this method in stomatal function models, e.g., for prediction of spatial variability in CO_2_ and water vapor fluxes.

### 4.2. Biological Significance of the Geometric Series of Stomatal Areas

The GS of stomatal areas may reflect the hierarchical regulation mechanism of stomatal development. For instance, the division or expansion of stomatal precursor cells may be constrained by a geometric progression rule, resulting in a fixed proportionate reduction in the area of subsequent stomata. This rule may be related to the optimization of epidermal cell space allocation, which is signified by a geometric distribution that can maximize the balance between stomatal density and size within a limited epidermal area, thereby coordinating photosynthetic efficiency and water use [13]. In fact, the spatial competition between stomata and adjacent epidermal cells tends to lead to a regular distribution of stomata at the areole level [14,36]. Moreover, the significant differences in the common ratio among different species (Figure 4A) may imply the differentiation of evolutionary adaptation strategies, such as drought-adapted species possibly optimizing stomatal response rates by adjusting the common ratio. Because the present study is only focused on the comparison of the two methods for testing the GS hypothesis, we did not examine the potential link of the differences in the common ratios across different species or different geographical population of the same species to abiotic and biotic factors. However, this merits further investigation. In addition, the numerical values of the common ratios of the 12 Magnoliaceae species, ranging between 1.001411 and 1.011223, are approximately equal to unity (Figure 4A), which suggests low inequality in size across stomata. This has also been validated by low Gini coefficients (quantifying the degree of inequality in stomatal area distributions) for the 720 micrographs [37].

## 5. Conclusions

The common ratio and the first term of a geometric series (GS) could be estimated using the nonlinear regression to fit the general term formula of the GS for each of the 720 stomatal area sequences of the 12 Magnoliaceae species. The mean absolute percentage error (MAPE) values between the observed and predicted cumulative stomatal areas were all smaller than 10%, and 99.6% (717 out of the 720 micrographs) were smaller than 5%. This further confirmed that the GS hypothesis applied to the stomatal area sequences. The new method developed (Method-2), based on nonlinear least squares fitting, had a better goodness of fit than the prior method (Method-1). Method-1 belongs to a stepwise estimation method and uses the mean of the common ratio and the mean of the first term as model parameters. Given that only 52.4% of the 720 MAPE values based on Method-1 were smaller than 5%, GS hypothesis was less strongly supported by the prior study. In addition, it is feasible to use the formula for the sum of the GS to calculate the total stomatal area per micrograph area with the estimated common ratio and the first term, as indicated by the very high coefficient of determination (*r*^2^) between the observed and predicted total stomatal areas for the 720 data sets that exceeded 0.9999. Thus, the present study provides a useful tool for calculating stomatal area per unit leaf area.

## Data Availability

The data can be found in the online Supplementary Table S1.

## Supplementary Materials

The following supporting information can be downloaded at https://www.mdpi.com/article/10.3390/plants14060893/s1: Table S1: Results of fitting the geometric series of stomatal area using the two methods.
